# 1. CHANGING THE LENS ON MENTAL HEALTH

**DOI:** 10.1093/schbul/sby014.000

**Published:** 2018-04-01

**Authors:** Alastair Campbell

**Affiliations:** Author

## Abstract

**Overall Abstract:** Alastair Campbell, talking from personal and family experience, and based on years of campaigning and study, urges a rethink of how we view mental health and mental illness.

